# Endoscopic ear surgery in the treatment of chronic otitis media with atelectasis

**DOI:** 10.1007/s00405-024-08845-0

**Published:** 2024-08-10

**Authors:** Giannicola Iannella, Annalisa Pace, Antonio Greco, Armando De Virgilio, Enrica Croce, Antonino Maniaci, Jerome R. Lechien, Federico Maria Gioacchini, Massimo Re, Giovanni Cammaroto, Tiziano Perrone, Salvatore Cocuzza, Giuseppe Magliulo

**Affiliations:** 1 Department of ‘Organi Di Senso’, University “Sapienza”, Viale Università 33, 00183 Rome, Italy; 2 Department of Otolaryngology, Kore University, Enna, Italy; 3 Faculty of Medicine and Pharmacy, University of Mons (UMons), Mons, Belgium; 4 Department of Clinical and Molecular Sciences, ENT Unit, Polytechnic University of Marche, Ancona, Italy; 5 Department of Head-Neck Surgery, Head-Neck and Oral Surgery Unit, Morgagni Pierantoni Hospital, OtolaryngologyForlì, Italy; 6 Otorhinolaryngology Unit, Civil Hospital, Alghero, Italy; 7 Department of Medical, Surgical Sciences and Advanced Technologies G.F. Ingrassia, University of Catania, Catania, Italy

**Keywords:** Atelectasis otitis media, Tympanic membrane retraction, Endoscopic ear surgery, Middle ear endoscopy

## Abstract

**Purpose:**

Atelectasis otitis media (AtOM) is a chronic condition where the tympanic membrane (TM) becomes retracted towards the middle ear and the ossicular chain. Surgical treatment for this condition could be indicated based on stage of atelectasis, patient’s clinical condition and hearing loss. Over the years, AtOM has been treated with various types of tympanoplasty under microscopic view. The aim of this study is to present the results of endoscopic ear surgery in AtOM.

**Methods:**

Forty-five patients who underwent endoscopic trans-canal tympanoplasty were included in the study. Preoperative features, intraoperative findings and postoperative outcomes were collected.

**Results:**

Preoperatively, none of the study's patients were classified with a Sadè Grade I, whereas grades II, III and IV were 3 (6.6%), 23 (32.1%) and 19 (67.8%) respectively. The 3 patients with Sadè grade II showed a conductive hearing loss higher than 20 dB and a continuous ear fullness, therefore they were surgically treated. The postoperative graft success rate was estimated at 95.5%. During follow-up, 2 patients showed a TM perforation (at 6 and 12 months after surgery) whereas 1 patient experienced a recurrence of atelectasis in the TM (16 months after surgery). The overall success rate at the final follow-up was calculated at 88.8%. The average preoperative air-conduction threshold was 51.1 ± 21.5, which reduced to 34.6 ± 22.1 (p = 0.04) at follow-up. The preoperative air–bone gap decreased from 28 ± 7.2 to 11.8 ± 10 (p = 0.002) after surgery.

**Conclusion:**

Atelectasis otitis media might be suitable for exclusive endoscopic surgical treatment, as it appears to exhibit a low recurrence rate and promising audiological outcomes.

## Introduction

Atelectasis otitis media (AtOM) is a chronic form of otitis media in which the tympanic membrane becomes retracted towards the middle ear and the ossicular chain. Tympanic membrane atelectasis can result from negative pressure in the middle ear associated with Eustachian tube dysfunction and/or a blockage of the middle ear ventilation pathways [1–5]. This middle ear condition could be further complicated by episodes of middle ear suppuration, conductive hearing loss, and progression toward conditions such as tympanosclerosis, cholesteatoma, or cholesterol granuloma [6–8].

There are numerous classification for the tympanic membrane atelectasis, but the most commonly used in clinical practice remains Sadè’s classification, which identifies 4 stages of tympanic membrane retractions [9–11]. Surgical treatment for this condition correlates with the stage of atelectasis and patient’s clinical condition. Surgery is typically indicated for stage 3 and 4, or for stages 1 and 2 presenting severe conductive hearing loss [1–6, 9, 10].

Historically, otitis media with atelectasis has been treated with various types of tympanoplasty under microscopic view [1–10]. In recent years, the emergence of Endoscopic Ear Surgery (EES) has transformed otologic surgery. The endoscope has been considered by many authors a suitable alternative to the microscope for treating middle ear diseases, including chronic otitis media or cholesteatomas [11–14]. However, definite information regarding surgical advancements, postoperative results, and guidance on when and for which patients or middle ear pathologies ESS should be preferred, continues to be a subject of ongoing discussion [15–17].

Endoscopes offer the advantage of a more adjustable line of sight, it can be moved closer to the surgical field, enabling an excellent view of middle ear structures and recesses. In addition, angled endoscopes allow further visualization of the bottom of retraction pockets and tissue invagination behind middle ear structures, which are classically much more challenging to observe using a conventional microscopic approach [18–21]. Based on this evidence and our experience on this ear disease, otitis media with atelectasis could be a suitable candidate for an exclusive endoscopic surgical treatment. Upon reviewing the current scientific literature, we observed that despite different authors have analyzed the outcomes of EES in chronic otitis media or cholesteatomas, there are few studies in the literature that have evaluated EES in patients with atelectasis otitis media [22–24].

The aim of this preliminary study is to present the use of endoscopic ear surgery in treating a group of adult patients with chronic otitis media with atelectasis. The outcomes, advantages and disadvantages of EES for treating this middle ear disease have been discussed.

## Materials and methods

### Trial design

This study was a single-center retrospective analysis of patients who suffered from chronic otitis media with atelectasis and underwent surgical treatment using an exclusive endoscopic surgical approach.

### Patient enrollment

Fifty-two patients, aged between 16 and 65 years, who underwent surgical treatment for chronic otitis media with atelectasis from January 2014 to January 2023, were initially reviewed and considered for study inclusion.

Exclusion criteria were the followings: incomplete preoperative or postoperative data, short or lost follow-up, identification of Epytimpanic or Mesotympanic cholesteatoma emerged during surgery, revision surgery cases and pediatric patients. Additionally, patients with normal hearing or a transmissive gap of less than 10 dB were not scheduled for surgery and thus not included in the study.

Following the application of exclusion criteria, forty-five patients (26 females and 19 males) who underwent endoscopic trans-canal tympanoplasty were included in the study. At the time of surgery, the patients’ ages ranged from 18 to 66 years (mean: 42.5 years). The surgery was performed on the right and left ears in 23 and 22 patients, respectively.

The medical history and otomicroscopic evaluation of the enrolled patients were collected.

**Fig. 1:**
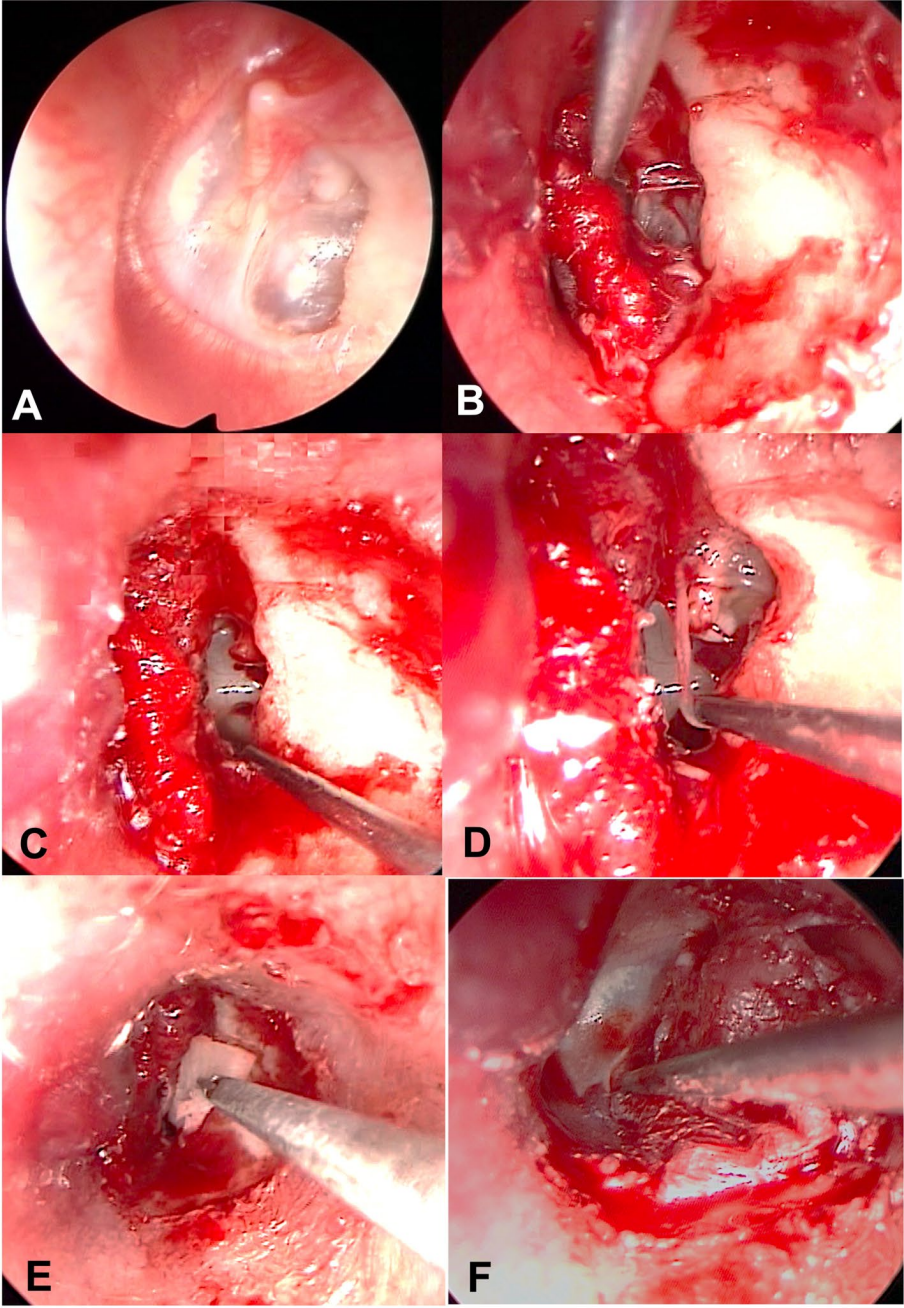
Endoscopic tympanoplasty for a grade III tympanic membrane retraction (Sadè classification). **A** The tympanic membrane is retracted and adherent to the incudo-stapedial joint. **B** Elevation of the tympano-meatal flap with the atelectasis tympanic membrane adhered to the ossicular chain. **C** The adhered tympanic membrane has been elevated, revealing the intact incudo-stapedial joint. The incus is non-eroded, and the ossicular chain remains intact. **D** Small atticotomy performed to explore the epitympanic region and open the tympanic isthmus. **E** Tympanic membrane graft using tragus cartilage + temporalis fascia in an underlay technique. **F** Final view of the reconstructed tympanic membrane. *i-s* incudo-stapedial joint, *a* atticotomy, *c* cartilage, *t-f* temporalis fascia

All patients were classified according to the Classification of tympanic membrane atelectasis proposed by Sadé:

- GRADE 1: Slight retraction of the tympanic membrane
- GRADE 2: Retraction of the tympanic membrane, touching the incus or stapes
- GRADE 3: Tympanic membrane retracted to the promontory but not adherent (Fig. 1A)
- GRADE 4: Tympanic membrane adherent to the promontory (adhesive otitis media) (Fig. 1B)

We used this classification system as it remains the most widely used to describe atelectasis and adhesive otitis media in adults.

### Surgery

Endoscopic ear surgery were performed using rigid endoscopes with 0°, 30° and 45° angulations, measuring 14 cm in length and with an outer diameter of 3 mm or 4 mm (Storz, Germany). The endoscopes were connected to a camera head (Storz, Germany) and a high-definition monitor positioned in front of the surgeon.

**Fig. 2:**
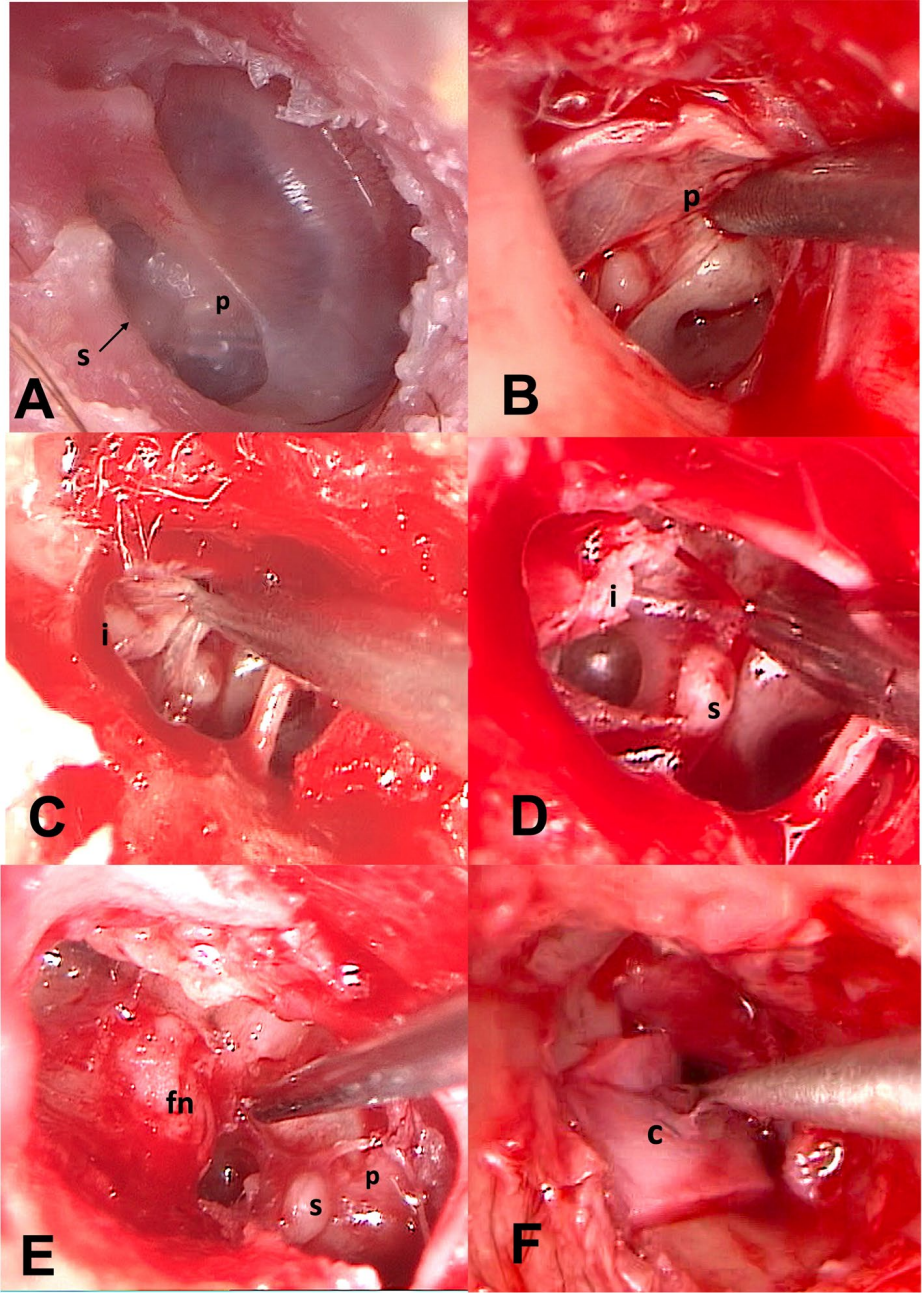
Endoscopic tympanoplasty for a grade IV tympanic membrane retraction (Sadè classification). **A** The tympanic membrane retracted and adherent to the promontory and stapes superstructure. **B** elevation of the tympano-meatal flap with atelectasis tympanic membrane tissue adhered to the stapes and promontory, eroded incudo-stapedial joint. **C** Removal of adhesion tissue around the stapes. **D** Atticotomy performed to explore the epitympanic region and remove tissue around the stapes and promontory. **E** Further atticotomy performed, removal of eroded incus, and all adhesive tissue. **F** Tympanic membrane graft using tragus cartilage + perichondrium. *s* stapes, *p* promontory, *i* incus, *fn* facial nerve, *c* cartilage

**Fig. 3:**
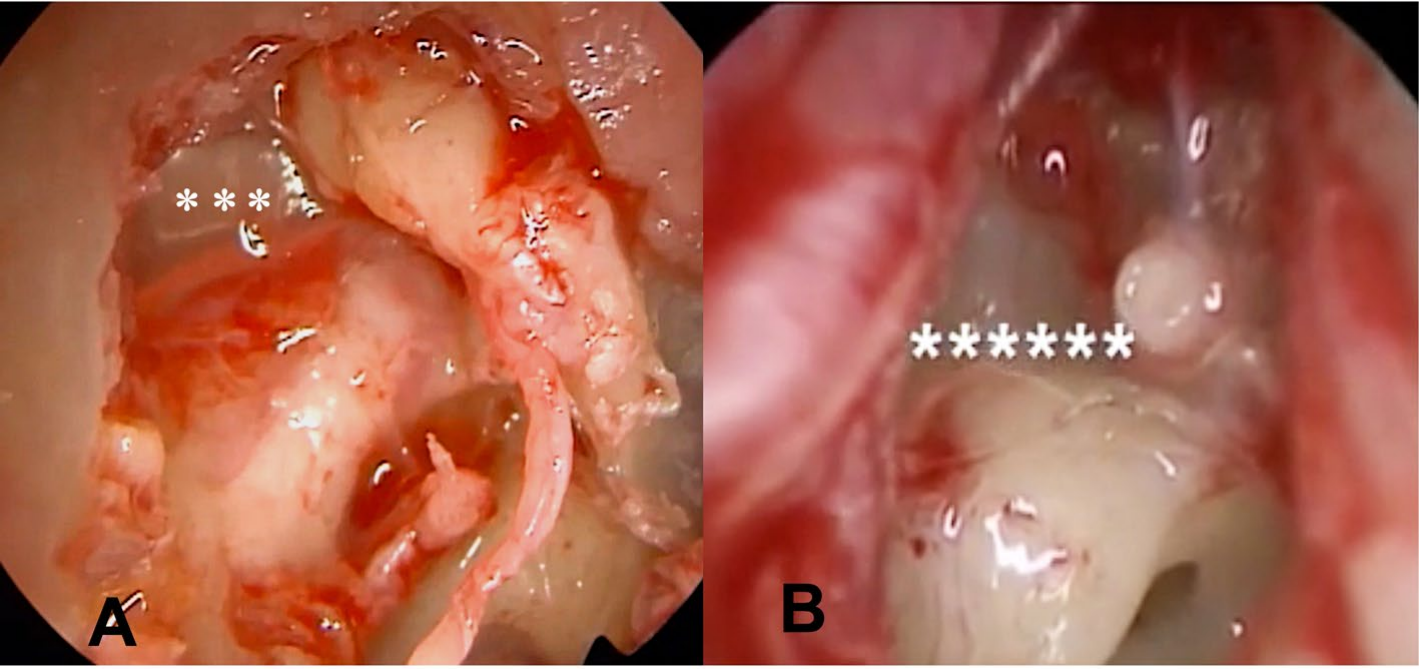
Endoscopic tympanoplasty. **A** Atticotomy performed, eroded incus removed. A blocked tympanic isthmus is evidenced. **B** Atticotomy performed and incus removed. A fold blocking the mesotympanic ventilation route is identified

The steps of EES consisted of:

- creation of the tympanomeatal flap in the posterosuperior and posteroinferior portions of the external auditory canal;
- access to the middle ear while preserving the chorda tympani; removal of the thinned tympanic membrane adhered to the promontory or incus/stapes (Fig. 1B, C; Fig. 2B, C);
- a small atticotomy in order to explore the epitympanic region if necessary (Fig. 1D; Fig. 2C, D);
- identification of the oval and round windows, the tympanic segment of the Fallopian canal, the cochleariform process and the horizontal semicircular canal (Fig. 2D, E);
- removal of any ossicles (incus and the head of malleus) if eroded (Fig. 2E);
- opening of the tympanic isthmus or tensor fold if blocked (Figs. 1D; 2E; 3A);
- graft with tragal cartilage ± temporalis fascia by using an underlay technique (Figs. 1E; 2F);
- repositioning of the tympanomeatal flap in its original position (Fig. 1F).

Avoiding a relapse of an AtOM requires the use of rigid grafts that can better resist negative middle ear pressure and can prevent the recurrence of retraction. Therefore, cartilage was used for tympanoplasty in our experience with AtOM treatment. Ossiculoplaty was not performed in case of incus or stapes supertructures erosion at the same time of the tympanoplasty surgery.

Each surgical procedure was performed using standard tympanoplasty instruments and recorded to enable subsequent review.

All surgical procedures were conducted by one of authors (G.M.) at the ‘Sensory Organs’ Department of the ‘Sapienza’ University of Rome.

Endoscopic surgery was introduced in our Otolaryngology division 18 months prior to the first patient’s surgical treatment with COM atelectasis.

### Intraoperative and postoperative evaluation

The intraoperative ossicles’ erosions were investigated in all patients enrolled in the study. The presence of facial nerve dehiscence was intraoperatively assessed by palpating the Fallopian canal bone during the surgery. The operative time was recorded, with the evaluation of the mean time.

Postoperative pain severity was assessed in all patients enrolled according to three grades: almost no pain, mild pain not requiring analgesic drugs and pain requiring analgesic therapy.

Taste abnormalities were investigated as: presence or absence of subjective abnormal taste sensations.

For patients experiencing postoperative dizziness, its duration in days was reported.

Follow-up visits were conducted approximately every 15 days for the initial 3 months post-surgery and then once every 3 months. The postoperative follow-up period ranged from 1.2 to 4.2 years (mean: 3.2 years).

Graft success was determined by assessing complete eardrum repair and the patient’s return to their normal activities. The healing time was also recorded.

Atelectasis recurrence or other middle ear complications/diseases (such as TM perforation or middle ear cholesteatoma) during patient follow-ups were evaluated. The overall success rate at follow-up was therefore calculated.

Hearing was assessed both preoperatively and at 1 month, 3 months, and 6 months after surgery. Final hearing recovery at six month follow-up was evaluated and classified according to the AAO-HNS draft hearing classification system [25].

#### Statistical analysis and ethical statement

A descriptive analysis was performed initially. Changes in data between preoperative and postoperative outcomes were evaluated using the Student's t-test via SAS, JMP8 version (SAS Institute, Cary, NC). A p-value of < 0.05 was considered the threshold for statistical significance. If no statistical significance was found between the two groups, a p-value > 0.05 was reported in the text. This research study adhered to the principles of the Declaration of Helsinki and received approval from the local Ethics Committee of the University “Sapienza” of Rome.

## Results

### Preoperative and intraoperative outcomes

Preoperative and intraoperative data for the enrolled patients are summarized in Table 1.

**Table 1:** Preoperative and intraoperative findings of the study group

Outcomes	EES (45 pts)
Sex	26 females–9 males
Average age	40.3 years
Preoperative symptoms	
Ear fullness sensation	41 (91.1%)
Otorrhea episodes	8 (19.04%)
Facial palsy	–
Vertigo/dizziness episodes	2 (4.4%)
Previous ventilation tube insertion	32 (71.11%)
Preoperative Sadè classification grade	
Grade I	–
Grade II	3 (6.6%)
Grade III	23 (32.1%)
Grade IV	19 (67.8%)
No ossicle erosion	26 (57.7%)
Incus erosion	14 (31.1%)
Incus + stapes superstructures erosion	5 (11.1%)
Block of tympanic isthmus	24 (53.3%)
Complete tensor fold	15 (33.3%)
Meso-pro tympanic fold	13 (28.8%)
Intraoperative facial nerve dehiscence	8 (17.7%)
Overall operation time (min)	72.8 min (high = 98.0 low = 59.0; standard deviation = 11.50)

Among the patients, 41 (91.1%) reported ear fullness, 8 (19.04%) experienced episodes of otorrhea, and 2 (4.4%) reported dizziness episodes.

Thirty-two (71.11%) of the surgically treated patients had previously undergone ventilation tube insertion to alleviate tympanic membrane atelectasis, without clinical success.

Prior to surgery, none of the patients were classified as Sadè Grade I. Grades II, III, and IV were identified in 3 (6.6%), 23 (32.1%), and 19 (67.8%) patients, respectively. The three patients with Sadè Grade II exhibited a conductive hearing loss higher than 20 dB and continuous ear fullness, leading to their surgical treatment.

Intraoperatively, no cholesteatoma pearls were found within the atelectasis of the tympanic membrane, and the preoperative Sadè classification was confirmed in all cases.

During surgery incus erosion was observed in 14 (31.1%) patients, while erosion involving both the incus and stapes superstructures was reported in 5 (11.1%) patients. However, no ossicular chain erosion was observed in 26 (57.7%) patients. There was no observable difference between the Sadè grade sub-classification and ossicular chain erosion within the study group (see Table 2).

**Table 2:** Intraoperative characteristics of ossicular chain erosion and ventilation routes blockage in atelectasic otitis media endoscopic surgical treated

	Grade 1I	Grade III	Grade IV	TOTAL	Statistical difference grade II vs grade III	Statistical difference grade III vs grade IV	Statistical difference grade II vs grade IV
Number of patients/percentage	3 (6.6%)	23 (51.1%)	19 (42.2%)	45			
No ossicular chain erosion	2 (66.6%)	15 (65.2%)	9 (47.3%)	26 (57.7%)	1	0.3	1
Incus erosion	1 (33.3)	6 (26%)	7 (36.8%)	14 (31.1%)	1	0.5	1
Incus + stapes superstructures erosion	–	2 (8.6%)	3 (15.7%)	5 (11.1%)	1	0.6	1
Block of tympanic isthmus	1 (33.3%)	12 (52.1%)	11 (57.8%)	24 (53.3%)	1	0.7	0.5
Complete tensor fold	–	8 (34.7%)	7 (36.8%)	15 (33.3%)	0.5	1	0.2
Meso-pro tympanic fold	–	7 (30.4%)	6 (31.5%)	13 (28.8%)	0.5	0.5	0.5
Intraoperative facial nerve dehiscence	–	4 (17.3%)	4 (21%)	8 (17.8%)	1	1	1

A blocked tympanic isthmus was observed in 24 (53.3%) cases, complete tensor fold in 15 (33.3%), and Meso-pro tympanic fold in 13 (28.8%) cases. No statistical difference was noted between Sadè sub-classifications and airway blockages. All folds obstructing middle ear airways were successfully reopened, restoring ventilation routes in all cases.

EES for atelectasis COM demonstrated an average surgical time of 72.8 min (high = 98.0, low = 59.0; standard deviation = 11.50).

### Postoperative outcomes

Postoperative outcomes has been reported in Table 3. The analysis of the postoperative pain revealed that 36 (80%) patients reported ‘no pain’ after surgery. Mild pain without needs of drugs was reported in 8 (17.7%) cases, whereas only 1 (2.3%) patient asked for pain medication.

**Table 3:** Postoperative outcomes of surgical treated patients

Outcomes	EES in atelectasis COM
Postoperative pain	
Almost no pain	36 (80%)
Mild pain no requiring analgesic drugs	8 (17.7%)
Pain requiring analgesic drugs	1 (2.3%)
Temporary postoperative dizziness	5 (11.1%)
Temporary abnormal taste sensation	8 (17.7%)
Graft success rate	43 (95.5%)
Average healing time (days)	29 ± 5.9 days
Middle ear disease at follow-up (atelectasis recurrence, TM perforation or middle ear cholesteatoma) (15.8 months of follow-up)	3 (6.6%—2 tympanic membrane perforations and 1 atelectasis recurrence)
Overall success rate at follow-up	40 (88.8%)
Failure of graft	2
atelectasis recurrence	1
TM perforation	2

Temporary Postoperative dizziness was reported in 5 (11.1%) cases. However, this symptom disappeared within 15 days post-surgery for all patients. Temporary abnormal taste sensation was present in 8 (17.7%) patients, which in all cases resolved within 3-months post-surgery.

Postoperatively, two patients showed issues with the temporalis muscle fascia graft attachment in the first month after surgery: one case involved tragus cartilage displacement towards the promontory, while the other patient showed cartilage necrosis. The graft success rate was estimated at 95.5%. During clinical follow-up, 2 patients experienced TM perforation (at 6 and 12 months post-surgery), and 1 patient had a recurrence of atelectasis in the TM (16 months post-surgery). The overall success rate at the final follow-up was calculated at 88.8%.

Hearing results pre- and post-surgery are presented in Table 4. The average preoperative air-conduction thresholds decreased from 51.1 ± 21.5 to 34.6 ± 22.1 at follow-up (p = 0.04). The preoperative air–bone gap reduced from 28 ± 7.2 to 11.8 ± 10 (p = 0.002).

**Table 4:** Audiological results pre and post endoscopic ear surgery for atelectasis otitis media

Average preoperative air-conduction thresholds (dB)	Average postoperative air-conduction thresholds (dB)	P-value (t student test)
51.1 ± 21.5	34.6 ± 22.1	0.04

Average preoperative air–bone gap	Average postoperative air–bone gap	
28 ± 7.2	11.8 ± 10	0.002

## Discussion

Managing Atelectasis otitis media poses significant challenges for otologists. The progression of retraction in this condition often leads to adherence of the atrophic membrane to the incus and stapes, increasing the risk of ossicle necrosis. Additionally, the TM may adhere to various parts of the middle ear, extending into the sinus tympani, superiorly into the attic, and inferiorly into the hypotympanum. Besides, retraction pockets in the tympanic membrane hold clinical significance as they contribute to the pathophysiology of middle ear cholesteatoma formation. Therefore, accurate diagnosis and proper management of these retraction pockets are crucial to prevent complete ossicle erosion and the formation of cholesteatoma [1–10].

Several authors advocate for conservative treatment in patients classified as Sadè Grade I and II or those with limited conductive hearing loss. Surgical intervention is often considered in Grade III or IV cases or in situations involving recurrent otorrhea or signs of progressive cholesteatoma [7–9].

Traditionally, atelectasis otitis has been managed through microscopic tympanoplasty using retro-auricular or trans-meatal approaches. However, in recent years, the endoscope has gained prominence in middle ear surgery [26–28]. The enhanced visualization provided by the endoscope, particularly the ability to view areas 'around the corner,' makes ESS an excellent approach for treating various middle ear disorders. Structures like the retrotympanum, anterior epitympanum, and middle ear folds, which are typically challenging to access, become more visible and accessible with endoscopic assistance [14–18]. While advantages and disadvantages of EES in chronic otitis media and cholesteatoma have been discussed in various papers, scientific evidence regarding the application of EES in atelectasis otitis media remains limited [22–24].

Marchioni et al. [24] conducted a preliminary study focusing on exclusive endoscopic tympanoplasty in patients with attic retraction pockets. They reported successful endoscopic dissection of the retraction pocket in all 27 subjects, removing the disease from the epi-tympanic region while preserving the mastoid bone and epitympanic-mastoid mucosa. Follow-up visits revealed a well-ventilated attic space in 21 of 27 (77.7%) subjects, with no retraction pockets found. In 5 of 27 (18.5%) subjects, a moderate retraction on the isthmus region was observed, maintaining good attic ventilation.

Guo et al. [23] presented a retrospective analysis of 17 patients with adhesive otitis media treated by EES. They reported excellent surgical outcomes, with all patients showing good tympanic membrane graft attachment, no retraction, or perforations observed at follow-up. The mean postoperative air-conduction hearing threshold (49.06 ± 22.15 dB hearing level) and mean air–bone gap (19.94 ± 10.00 dB HL) were significantly improved compared to preoperative values (65.29 ± 21.53 and 32.53 ± 8.21 dB HL, respectively; p < 0.05). No recurrences, secondary cholesteatomas, or secondary surgeries were reported at follow-up.

Parab et al. [22] described the treatment of 41 patients with retractions pockets surgically treated using two handed trans-canal endoscopic cartilage tympanoplasty with endoscope holders. At the 3-year clinical follow-up, no recurrence of retraction was observed. The preoperative air–bone gap was 24.53 ± 4.326 dB, improving to 14.57 ± 3.88 dB at the same follow-up period.

Despite these limited studies, there remains a lack of comprehensive investigations with larger sample sizes evaluating the surgical effectiveness of oto-endoscopic tympanoplasty for adhesive otitis media. Our study aims to contribute to the current understanding and effectiveness of ESS in managing atelectasis otitis media [29, 30].

In our study, ESS achieved an estimated graft success rate of 95.5% for treating atelectasis otitis media. At clinical follow-up, 2 patients experienced TM perforation (at 6 and 12 months post-surgery), and 1 patient had a recurrence of atelectasis TM (16 months after surgery), resulting in an 88.8% success rate at final follow-up. The average preoperative air-conduction thresholds decreased from 51.1 ± 21.5 to 34.6 ± 22.1 (p = 0.04) at follow-up, while the preoperative air–bone gap reduced from 28 ± 7.2 to 11.8 ± 10 (p = 0.002). These data closely align with the findings described by Parab et al. [22] and Guo et al. [23] reported above. The analysis of the postoperative pain showed as 36 (80%) of the patients reported ‘no pain’ after surgery, a quite high percentage.

In contemporary literature, authors have documented a lack of recurrence in 67–74% of patients with grade III and IV retraction pockets who undergo tympanoplasty using a microscopic approach. Interestingly, these statistics closely resemble those previously reported regarding the use of Endoscopic Ear Surgery (EES) for adhesive otitis media (AtOM).

**Fig. 4:**
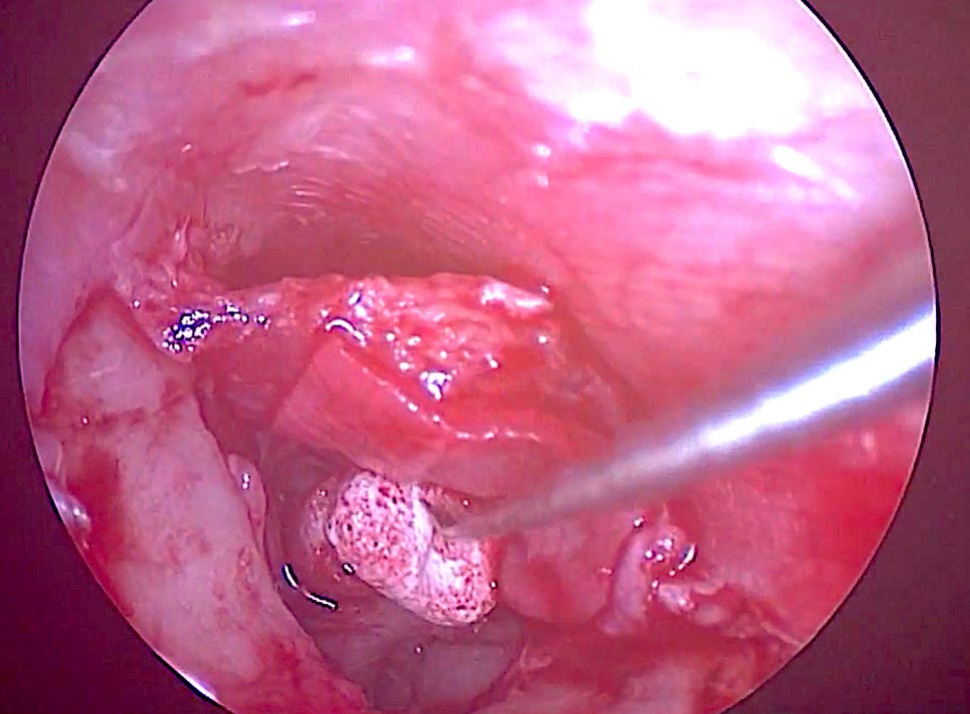
Ossicular chain reconstruction using PORP. Ossiculoplasty with PORP in secondary planned surgery. The patient previously underwent tympanoplasty for chronic otitis media with atelectasis, during which the eroded incus was removed. Stapes superstructures were intact, and the footplate was present and mobile. The prosthesis is first placed with the hollow portion on the stapes head and then positioned under the TM in the correct position

In patients with tympanic membrane atelectasis where we remove the eroded incus or stapes superstructures, ossiculoplasty was not performed at the same time of the tympanoplasty surgery. In these patients an ossicular chain reconstruction using a TORP or PORP through endoscopic ossiculoplasty was scheduled 1 year after surgery (Fig. 4).

The primary advantages of EES lie in its excellent visualization of middle ear structures and its ability to explore structures around corners. Several anatomical and physiological concepts related to the development of Adhesive Otitis Media (AtOM) due to middle ear dysventilation align with the advantages offered by ESS. The blockage of ventilation routes by middle ear folds can contribute to epitympanic dysventilation and tympanic membrane retraction [12–18]. Therefore, it's crucial during surgery to ensure proper intraoperative visualization and the removal of these folds that obstruct airflow (Fig. 3). According to Marchioni et al. [24], the endoscopic procedure facilitates improved visualization and removal of blockages in the isthmus and the complete sectioning of the tensor fold, thereby creating a well-ventilated epitympanic cavity. The authors advocate that these endoscopic interventions, focusing on opening blocked airways while preserving the mastoid, could play a vital role in preventing a recurrence of attic retraction.

Despite offering excellent visualization of middle ear structures and recesses, the endoscopic approach has some potential limitations. These include the challenge of single-handed work, the absence of a stereoscopic view, and a potentially steep surgical learning curve. Additionally, the need for frequent endoscope cleaning due to blood or bone dust in the surgical field poses risks, including potential injury to middle ear structures during manipulation of the tympanic membrane adhered to the ossicular chain [14–19].

Furthermore, when compared to microscopic surgery, EES demonstrates less effective management of surgical complications, such as trauma to vital structures [15–18]. These factors collectively contribute to the relatively limited role played by exclusive Endoscopic Ear Surgery (EES) in comparison to conventional Microscopic Ear Surgery (MES).

## Conclusions

Otitis media with atelectasis might find suitability for exclusive endoscopic surgical treatment, as it seems to show a low recurrence rate and promising audiological outcomes. The utilization of an endoscope shows promise in reinstating ventilation pathways within the middle ear, particularly in cases where these routes are obstructed.

However, to comprehensively discern the nuances and weigh the advantages and disadvantages between Endoscopic Ear Surgery (ESS) and Microscopic Ear Surgery (MES) for treating atelectasis otitis media, further prospective comparative clinical studies are essential. These studies would serve to elucidate the differences and potential benefits or drawbacks associated with these two distinct surgical approaches, guiding clinicians towards optimal treatment strategies.
