# Correction: Endoscopic ear surgery in the treatment of chronic otitis media with atelectasis

**DOI:** 10.1007/s00405-024-08962-w

**Published:** 2024-09-16

**Authors:** Giannicola Iannella, Annalisa Pace, Antonio Greco, Armando De Virgilio, Enrica Croce, Antonino Maniaci, Jerome R. Lechien, Federico Maria Gioacchini, Massimo Re, Giovanni Cammaroto, Tiziano Perrone, Salvatore Cocuzza, Giuseppe Magliulo

**Affiliations:** 1 Department of ‘Organi Di Senso’, University “Sapienza”, Viale Università 33, 00183 Rome, Italy; 2 Department of Otolaryngology, Kore University, Enna, Italy; 3 Faculty of Medicine and Pharmacy, University of Mons (UMons), Mons, Belgium; 4 Department of Clinical and Molecular Sciences, ENT Unit, Polytechnic University of Marche, Ancona, Italy; 5 Department of Head-Neck Surgery, Head-Neck and Oral Surgery Unit, Morgagni Pierantoni Hospital, OtolaryngologyForlì, Italy; 6 Otorhinolaryngology Unit, Civil Hospital, Alghero, Italy; 7 Department of Medical, Surgical Sciences and Advanced Technologies G.F. Ingrassia, University of Catania, Catania, Italy

**Correction: European Archives of Oto-Rhino-Laryngology**

10.1007/s00405-024-08845-0

In this article the author’s name Federico Maria Gioacchini was incorrectly written as Francesco Maria Gioacchini. The original article has been corrected.

